# Effectiveness of Exercise Interventions to Improve Postural Control in Older Adults: A Systematic Review and Meta-Analyses of Centre of Pressure Measurements

**DOI:** 10.1007/s40279-016-0559-0

**Published:** 2016-05-31

**Authors:** Daniel C. Low, Gregory S. Walsh, Marco Arkesteijn

**Affiliations:** Institute of Biological, Environmental and Rural Sciences (IBERS), Aberystwyth University, Carwyn James Building, Penglais Campus, Aberystwyth, SY23 3FD UK

## Abstract

**Background:**

Previous reviews have shown balance in older adults to be improved with exercise. However, it is currently unclear whether postural control, indicated by centre of pressure (COP) measurement, can be improved in older adults and thus whether postural control could be a mechanism to improve balance.

**Objectives:**

The purpose of this systematic review was to assess the effectiveness of force platform COP variables to identify changes in postural control following exercise interventions in older adults. In addition, a secondary purpose was to determine whether the exercise types (balance, resistance or multi-component exercise interventions) are equally effective to improve postural control.

**Methods:**

Randomised controlled trials were identified using searches of databases and reference lists (PROSPERO registration number CRD42014010617). Trials performing exercise interventions, reporting force platform COP measurements, in participants with a mean age of ≥60 years were included. Risk of bias assessments were performed following the Cochrane guidelines. Data were pooled in meta-analyses, and standardised mean differences (SMDs) with 95 % confidence intervals (CIs) were calculated.

**Results:**

Twenty-three trials met the inclusion criteria for the systematic review. Twenty-two trials could be defined as either utilising a balance, resistance or multi-component exercise intervention. These 22 trials were used in the meta-analyses. All trials reported measurements of double leg stance; eight trials reported additional stance conditions. The meta-analyses of double leg stance showed that balance exercise interventions significantly decreased total sway path length/velocity [SMD −1.13, 95 % CI −1.75 to −0.51 (eyes open); SMD −0.79, 95 % CI −1.33 to −0.26 (eyes closed)] and anterior-posterior sway path length/velocity [SMD −1.02, 95 % CI −2.01 to −0.02 (eyes open); SMD −0.82, 95 % CI −1.46 to −0.17 (eyes closed)] in both eyes open and eyes closed conditions. Balance exercise interventions also decreased sway area in eyes closed conditions (SMD −0.57, 95 % CI −1.01 to −0.13) and medio-lateral sway path length/velocity in eyes open conditions (SMD −0.8, 95 % CI −1.48 to −0.12). In contrast, neither resistance nor multi-component exercise interventions affected any of the included COP measurements.

**Conclusions:**

Postural control is improved by balance exercise interventions. In contrast, strength or multi-component exercise interventions did not influence postural control measurements in older adults. In addition, a lack of standardisation in collection protocol and COP variables calculated across trials was identified.

**Electronic supplementary material:**

The online version of this article (doi:10.1007/s40279-016-0559-0) contains supplementary material, which is available to authorized users.

## Key Points

**Table:** 

Balance exercise interventions improve postural control in older adults.
Strength and multi-component exercise interventions do not improve postural control in older adults.
Study protocols showed large variations across the included studies, which likely affected the heterogeneity of the meta-analyses. It is recommended that future trials standardise postural control assessment, by employing the double leg stance, preferably with eyes closed, and a minimum duration of 60 s and at least three to five trials.

## Introduction

One third of adults aged over 65 years and half of adults aged over 80 years will experience a fall [1]. The aetiology of falls is multi-factorial and can be associated with age-related muscle weakness, impaired balance and gait, visual impairment and sex [1]. Further still, a single fall for an older adult increases the risk of recurrent falls and often causes a greater fear of re-falling, which can become debilitating [1]. Falls prevention programmes are often multi-factorial, with exercise being regarded as an essential component, specifically focusing towards balance improvement [2].

Systematic reviews have shown that exercise interventions can improve physical function, balance and potentially reduce the risk of falls [3–5]. Quantifying falls incidence is complex, requiring longitudinal monitoring, and often relies on subjective feedback from participants. Consequently, longitudinal monitoring is less common than balance assessments that act as indicators of falls risk [6]. Balance assessments can be performed statically (e.g. single leg stance, tandem stance) or dynamically (e.g. Tinetti’s Gait and Balance Assessment and the Berg Balance Scale) [7]. A recent review has, however, suggested that the ability for exercise interventions to improve static balance performance in frail older adults is inconsistent [7]. A potential explanation is that the outcome measure for functional and static balance assessments is commonly performance time, while dynamic tasks require a rating score; both have ceiling effects in healthy older adults [8]. Inclusion of postural sway measurements taken during the static tasks provides additional information regarding the health of the postural control system [9].

Pollock et al. [9] define postural control as ‘the act of maintaining, achieving or restoring a state of balance during any posture or activity’. It thus represents the body’s ability to respond to visual, vestibular and proprioceptive input with voluntary muscular output to maintain a stable upright posture and prevent a fall from occurring [9]. Several studies have demonstrated a positive association between impaired postural control and fall risk in older adults [10]. A force platform can be used to measure the extent of change in postural control following an exercise intervention [11]. However, the assessment protocols can be varied and include a multitude of outcome variables to indicate the individual’s degree of postural control [4]. The sway area and the sway path length are two common outcome variables that are calculated from force platform data, and have been associated with prospective falls [12, 13]. Previous systematic reviews have evaluated the effect of exercise intervention on older adults for functional measures of balance rather than postural control [3, 4] or have only reviewed balance training and failed to investigate the effect of the different study protocols (e.g. single leg and double leg stance) [5]. Thus, there is currently no systematic review focusing specifically on the effect of exercise interventions on these indicators of postural control.

The objective of the systematic review was to determine whether balance, strength and multi-component exercise intervention could improve postural control of older adults with a mean age of ≥60 years. The aim of the systematic review was to identify all randomised control trials involving older adults that measured postural control using a force platform before and after undertaking an exercise intervention. Subsequently, the effectiveness of different force platform measurements to indicate a change in postural control following exercise interventions was determined.

## Methods

### Literature Search and Eligibility Criteria

The systematic review protocol was published on the PROSPERO Register of Systematic Reviews (registration number CRD42014010617). The systematic review was designed collectively by the three members of the review team following the Cochrane PICO guidelines [14]. A systematic search for randomised controlled trial design studies was performed. For crossover randomised controlled trials, only data for the initial collection period was included. The following databases were searched: ISI Web of Knowledge, PubMed, Science Direct, Cochrane Controlled Trials Register, Proquest, LILACS, National Institutes of Health (NIH), NIH Research Portfolio Online Reporting Tools (RePORT) and metaRegister of Controlled Trials. Each database was searched using the terms identified in Table 1 for all available years up to 5 June 2015. A hand search of reference lists of the identified studies was also performed.

**Table 1:** A list of systematic review search terms following the PICO guidelines

	Terms used
Population	Elderly OR aging OR ageing OR old* OR geriatric
Intervention	Exercise OR training OR power OR balance OR flexibility OR strength OR resistance OR tai chi OR intervention
Comparison	Centre of pressure OR center of pressure OR COP OR postural sway OR postural stability OR postural control OR balance OR force plat*
Outcome	Randomised control* OR randomized control* OR RCT AND falls OR falling OR faller*

Eligible studies included participants with a mean age of ≥60 years. The participants’ health status was not an exclusion criterion. Included interventions were those that consisted of exercise activities, defined as any intervention of which the primary component (allowing for multi-component interventions) was structured physical activity (e.g. tai chi training). When different exercise interventions were compared against a control, the data for the exercise task most used in other research studies were extracted. The control group could consist of a non-exercise group (maintaining usual activity level), usual health care, or educational activities. Studies were included if the outcome measure quantified the magnitude of the centre of pressure (COP) movement (sway area, sway path length and sway velocity) and was derived with the use of a force platform. Indirect measures of balance (e.g. single leg stance time, Berg Balance Scale, timed up and go) were excluded, as they do not quantify postural control.

Two reviewers screened the titles and abstracts of all database results to identify relevant trials; discrepancies were resolved by consensus with a third reviewer. Full-text articles of all relevant studies were retrieved and submitted to the same review process of the inclusion criteria. Studies selected for review were limited to those written in English.

### Data Extraction

Details of the exercise intervention taken for analysis included the type of exercise, session frequency, duration and intensity, and the intervention duration. The age, sex, health status and sample sizes for both the intervention and control groups were also recorded. Similarly, the type of static balance assessment (e.g. double leg stance with eyes open) and details regarding the duration, frequency and data recording characteristics (e.g. sampling frequency and post-processing) were included in the extracted data. The primary outcome data taken and used in the analyses included the post-intervention mean and standard deviations with units for the COP variables. Authors were contacted to obtain missing data or data that were only presented graphically. Where possible, data that were not available as mean and standard deviation were derived through scaling of graphical representations. Data were extracted independently and were then checked by the two other members of the review team.

### Quantitative Synthesis

All analyses were performed using RevMan software (Review Manager 5.3, The Nordic Cochrane Centre: Copenhagen, The Cochrane Collaboration, 2014). The following COP variables were used in meta-analyses: sway area and total, medio-lateral (ML) and anterior-posterior (AP) path lengths and velocities, in two postural conditions (double leg stance with eyes open and with eyes closed). For meta-analyses, length and velocity variables were combined to improve statistical power, as these two variables are analogous mathematically, due to the constant trial times within studies. Sway path length was selected when trials reported both sway path length and velocity variables. Analyses were performed for each variable under each condition using a random effects model of standardised mean differences with 95 % confidence intervals (CI), and two-tailed *p* values were calculated. Meta-analyses were performed separately for three classifications of intervention type, consisting of balance, resistance, and multi-component exercise interventions. Heterogeneity was indicated using the Chi^2^ and *I* ^2^ statistics. A significant Chi^2^ (*p* < 0.05) and *I* ^2^ greater than 75 % represented considerably heterogeneous data [14].

### Risk of Bias Assessment

Two reviewers assessed each included trial for risks of bias independently, using the Cochrane risk of bias assessment tool [14]. Briefly, this tool covers seven domains: sequence generation, allocation concealment, blinding, blinding of outcome assessment, incomplete outcome data, selective outcome reporting, and other issues. Each domain is subjectively scored as high, low or unclear risk if insufficient information is provided. Discrepancies were resolved by consensus. No studies were excluded following the risk of bias assessment.

## Results

### Search Results

**Fig. 1:**
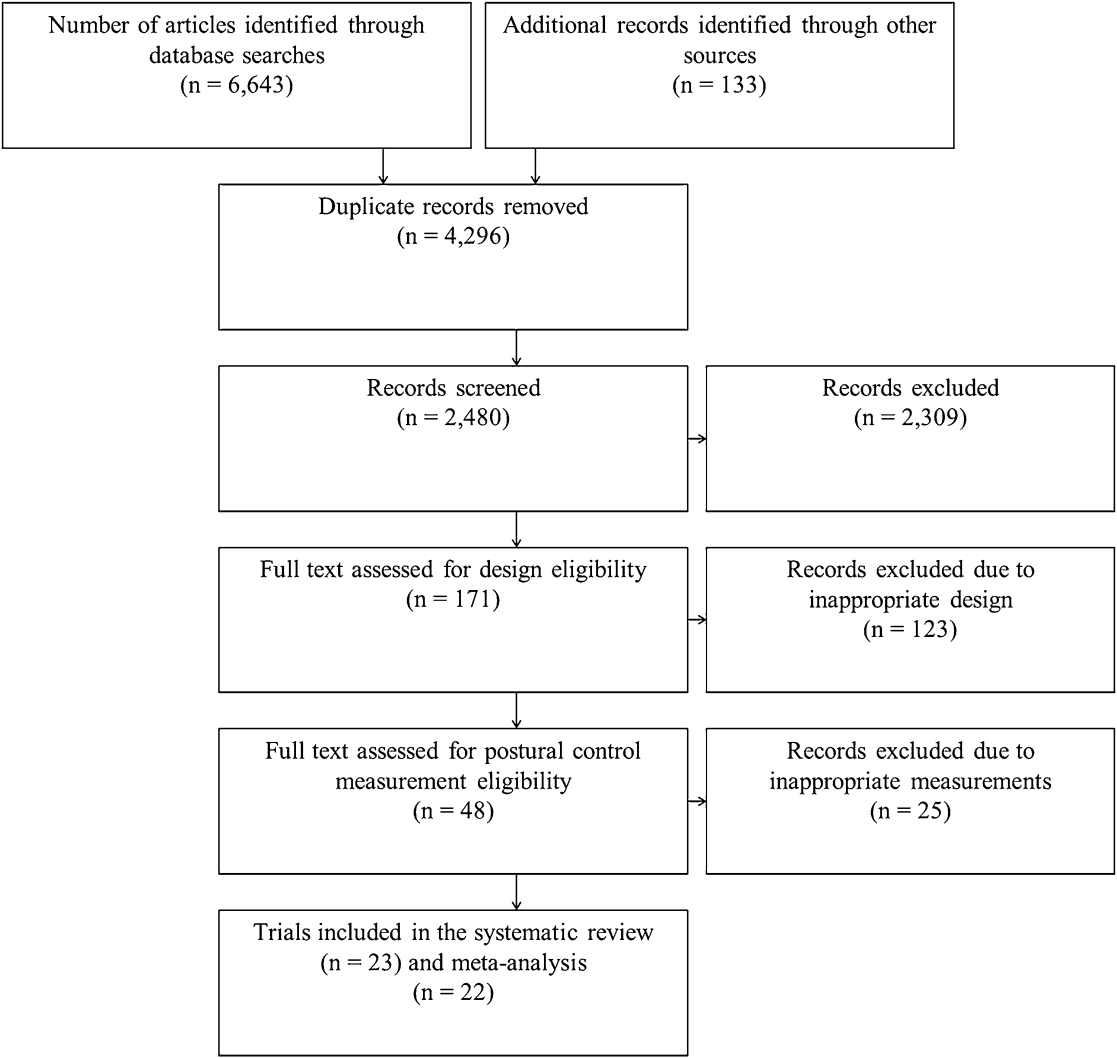
Detailed flow of studies through the systematic review

A detailed flow of studies through the review is shown in Fig. 1. In summary, the search strategy produced a total of 6776 studies. After the removal of duplicates and the screening of the abstracts and full text for appropriate design or postural control assessment method, 22 trials were included in the meta-analyses. Please see the Electronic Supplementary Material Tables S1 and S2 for full study details.

### Risk of Bias Assessment

Table 2 demonstrates the risk of bias assessment performed on the selected papers used in the meta-analyses.

**Table 2:** Risk of bias assessment for papers used in the meta-analysis

References	Sequence generation	Allocation concealment	Blinding of researchers	Incomplete outcome data	Selective outcome reporting	Other sources of bias
Álvarez et al. [17]	LRB	LRB	LRB	LRB	UCB	LRB
Crilly et al. [16]	LRB	UCB	UCB	LRB	UCB	LRB
del Pozo-Cruz et al. [20]	LRB	UCB	LRB	LRB	UCB	LRB
de Oliveria et al. [23]	LRB	UCB	LRB	LRB	UCB	LRB
Donath et al. [34]	LRB	UCB	UCB	LRB	UCB	LRB
Elbar et al. [32]	LRB	LRB	LRB	LRB	UCB	LRB
Hiyamizu et al. [35]	LRB	LRB	LRB	LRB	UCB	LRB
Judge et al. [24]	UCB	UCB	UCB	LRB	UCB	LRB
Kaneda et al. [33]	UCB	UCB	LRB	HRB	UCB	LRB
Katsura et al. [27]	UCB	UCB	UCB	LRB	UCB	LRB
Lai et al. [29]	UCB	UCB	LRB	LRB	UCB	LRB
Lee and Song [19]	LRB	UCB	LRB	LRB	UCB	LRB
Lee et al. [22]	UCB	UCB	UCB	HRB	UCB	LRB
Lelard et al. [25]	UCB	UCB	UCB	UCB	UCB	LRB
Nagai et al. [30]	LRB	HRB	HRB	LRB	UCB	LRB
Nagy et al. [36]	UCB	UCB	UCB	LRB	UCB	LRB
Nicholson et al. [31]	LRB	UCB	LRB	LRB	UCB	LRB
Ni et al. [15]	UCB	UCB	UCB	LRB	UCB	LRB
Park et al. 2012 [28]	LRB	LRB	LRB	LRB	UCB	LRB
Pluchino et al. [26]	LRB	LRB	UCB	LRB	UCB	LRB
Song et al. [21]	UCB	UCB	UCB	LRB	UCB	LRB
Yennan et al. [18]	UCB	UCB	UCB	LRB	UCB	LRB
Overall, *n* (%)	LRB 12 (54.5)	LRB 5 (22.7)	LRB 10 (45.5)	LRB 19 (86.4)	LRB 0 (0)	LRB 22 (100)
	HRB 0 (0)	HRB 1 (4.5)	HRB 1 (4.5)	HRB 2 (9.1)	HRB 0 (0)	HRB 0 (0)
	UCB 10 (45.5)	UCB 16 (72.7)	UCB 11 (50.0)	UCB 1 (4.5)	UCB 22 (100)	UCB 0 (0)

*HRB* high risk of bias, *LRB* low risk of bias, *UCB* unclear risk of bias

### Population Characteristics and Exercise Intervention Protocols

Of the 23 trials included in the systematic review, 15 were conducted using a healthy community-dwelling population. The remaining eight studies consisted of fallers [15], institutionalised [16, 17], osteoarthritic [18], diabetic [19, 20] and diabetic peripheral neuropathic [21, 22] populations. Four trials included only women [16, 18, 23, 24]. Eight trials included in the review did not use a non-exercise control group where usual activity level was maintained. These included controls participating in balance [15, 25, 26], flexibility [24] strength [18] or altered versions of the experimental intervention [23, 27, 28].

The most common single component exercise intervention was balance training, of which six trials incorporated regular balance exercise [19, 21, 22, 29–31] and three performed tai chi [15, 25, 26]. Eight trials utilised resistance exercise tasks, whereby five were water based [18, 23, 27, 32, 33], two used vibration [17, 20], and one trial evaluated stair climbing [34] exercises. Five trials employed multi-component interventions, including strength and balance [16, 35, 36], strength and tai chi [24], and therapeutic exercises and jumping [28]. One trial evaluated walking exercises [37] and could not be classified as a balance, resistance or multi-component intervention and thus was not included in any meta-analyses.

### Postural Control Measurement

There was disparity evident in the measurement protocol used to record COP. The number of trials for each condition ranged from one [29, 30, 33, 35, 36] to ten [32], and trial duration ranged from 8 s [24] to 75 s [29]. The force platform or measurement equipment sampling frequency ranged from 5 Hz [28] to 1000 Hz [17], but was not reported by nine trials [15, 16, 18, 19, 21, 22, 26, 27, 33]. Seven studies reported the use of a digital filter on the COP signal [20, 23, 30, 31, 34, 36, 37].

### Postural Control Conditions

All included trials reported at least one measurement with eyes open, and 18 reported at least one measurement with eyes closed (Table 2). All trials performed measurements in double leg stance, and eight trials performed measurements in single leg [18, 23, 24, 34], narrow [20, 31] and semi-tandem [23, 37]. One trial performed double leg stance on a foam surface [37], and two trials reported measurements collected during dual tasking [17, 35].

### Postural Control Variables

The most common COP variable reported was the total sway path length [15, 16, 19–21, 24–27, 33–35]. Ten trials reported measures of sway area [15, 17, 18, 23, 25, 26, 29, 30, 32, 33], ten trials reported ML sway length [16, 18–21, 28, 31, 32, 36, 37] and nine trials reported AP sway length [16, 18–21, 28, 32, 36, 37]. Total path velocity was reported by five trials [15, 25, 26, 29, 37], and three trials reported AP [22, 23, 37] and ML [22, 23, 37] path velocity, respectively.

### Effect of Exercise Type on Postural Control

**Fig. 2:**
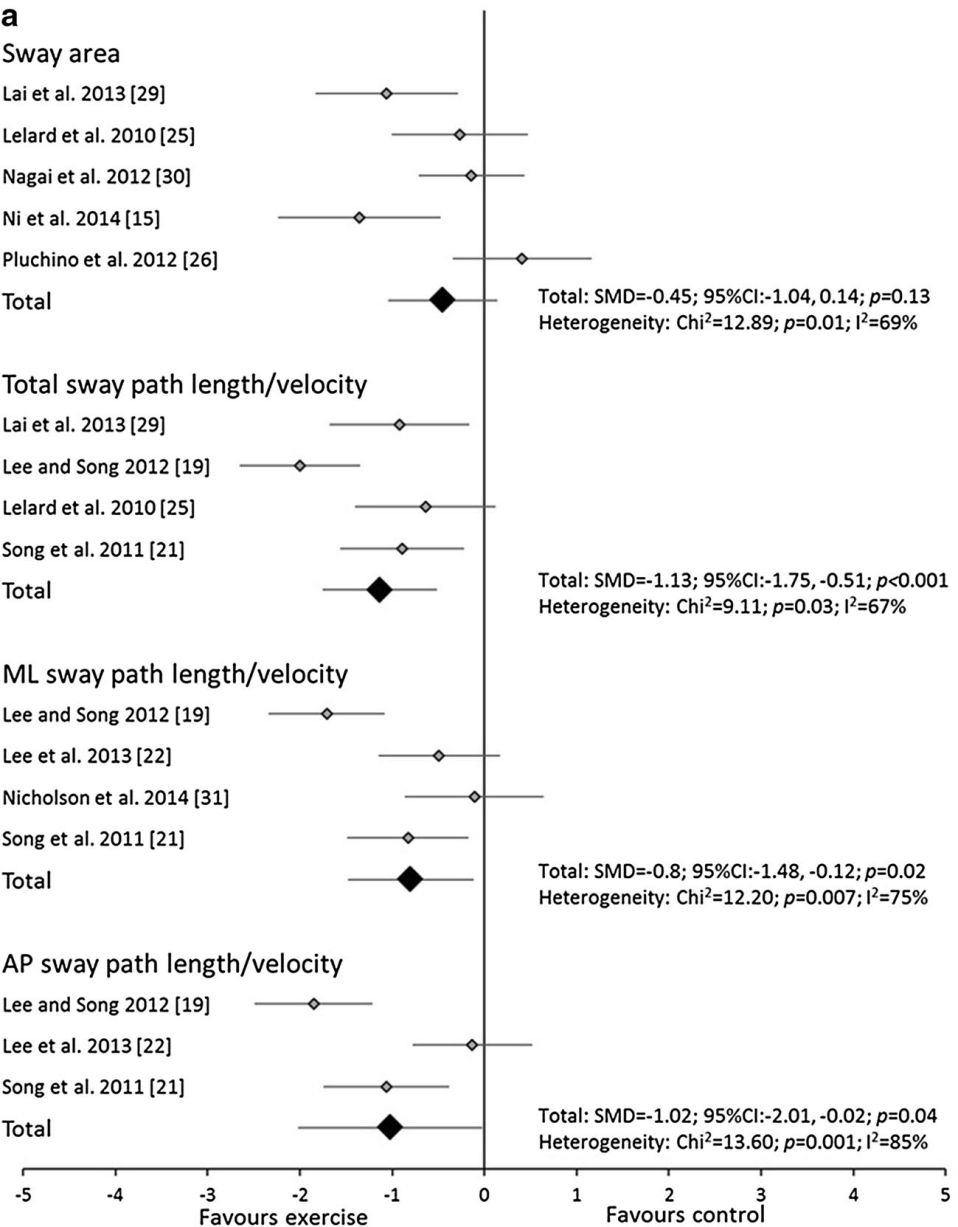

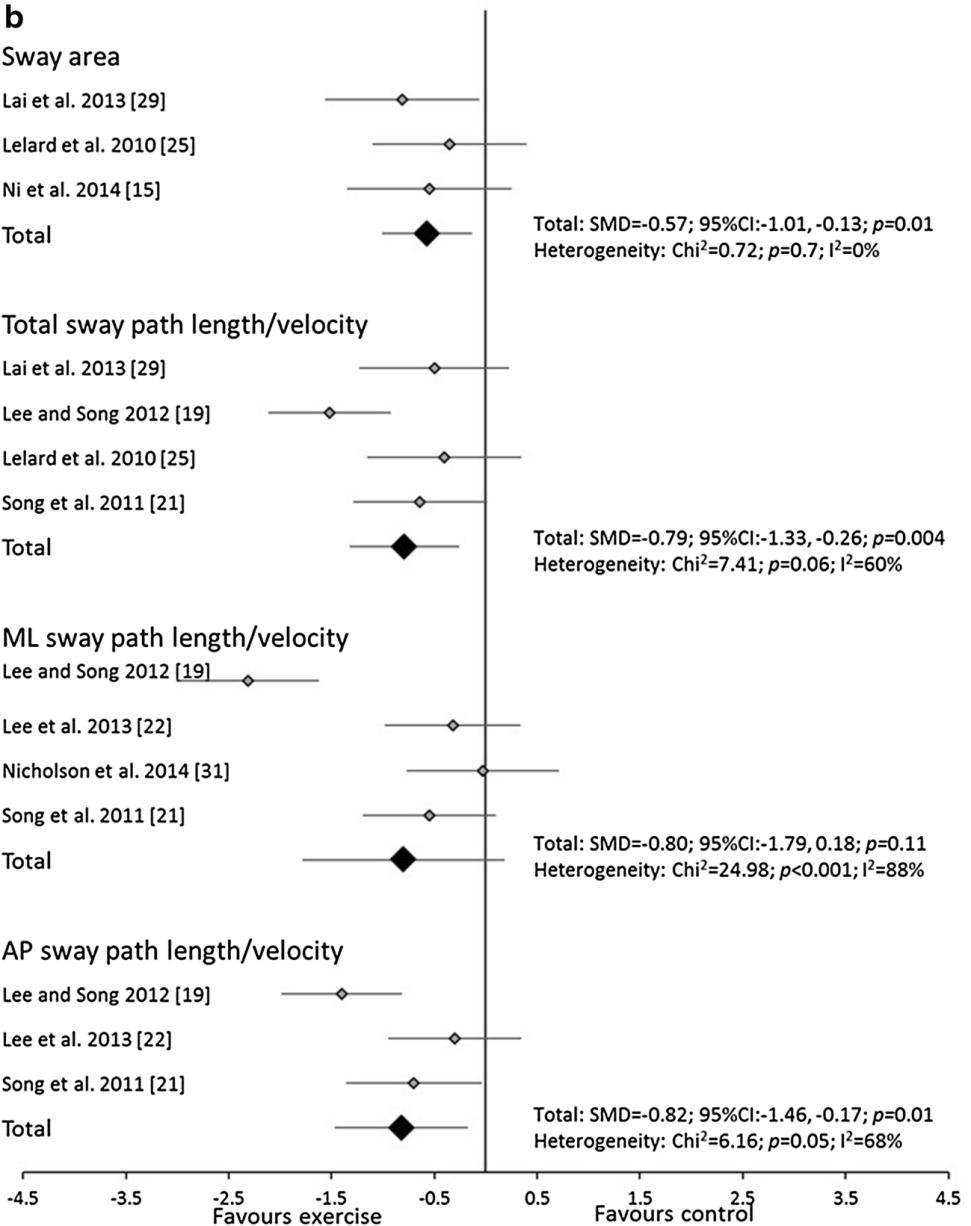
Forest plot of standardised mean differences (SMDs) and 95 % confidence intervals (CIs) for sway area and total, medio-lateral (ML) and anterior-posterior (AP) sway path length/velocity under **a** eyes open and **b** closed, double leg stance conditions for the balance training interventions

The meta-analyses utilised a total of 22 trials. The analyses revealed that balance exercise interventions significantly decreased total sway path length/velocity and AP sway path length/velocity in both eyes open and eyes closed conditions. Balance exercise interventions also decreased sway area in eyes closed conditions and ML sway path length/velocity in eyes open conditions (Fig. 2a, b). Significant heterogeneity was observed for most variables except sway area and total and AP sway path length/velocity in eyes closed conditions for balance exercise interventions.

**Fig. 3:**
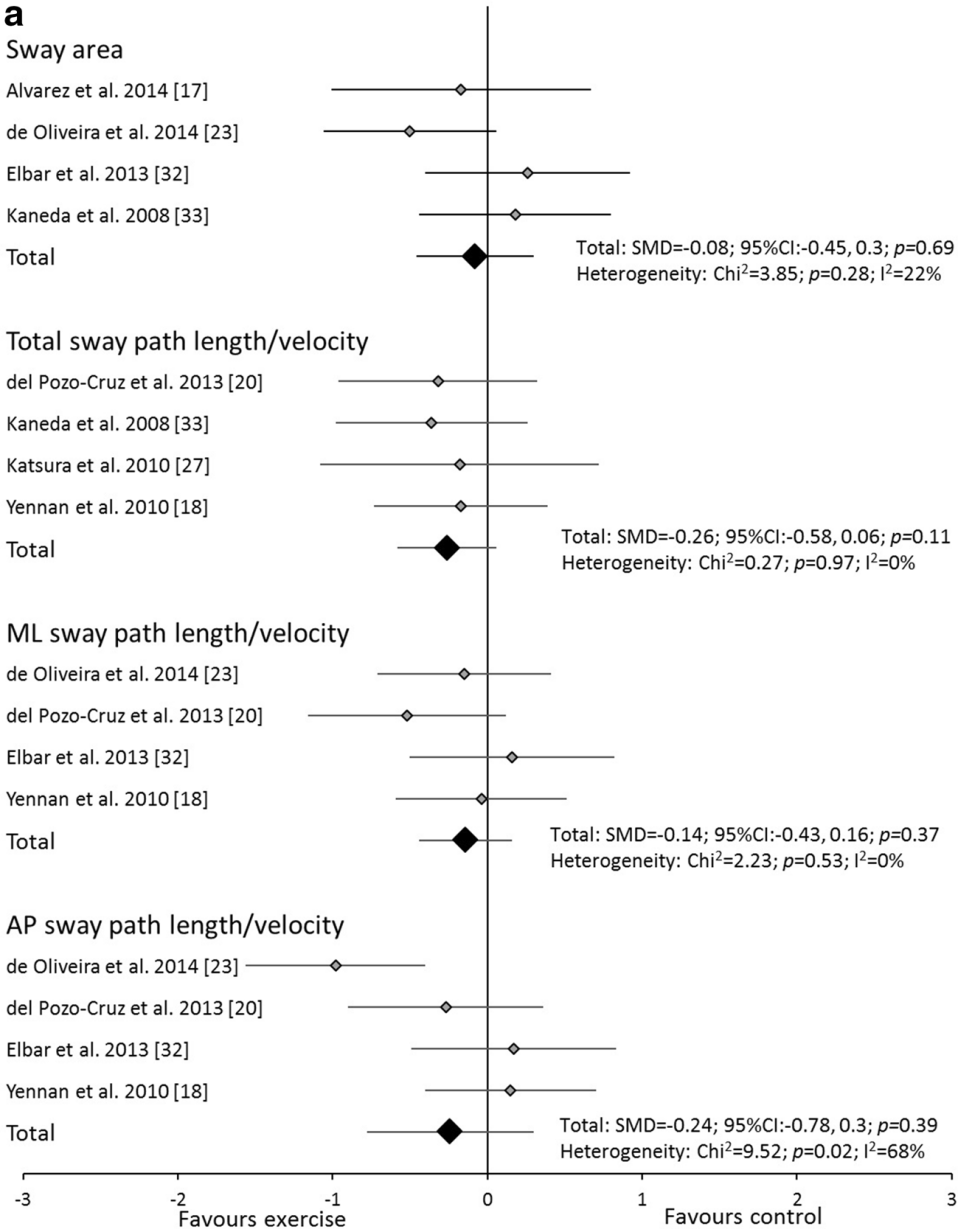

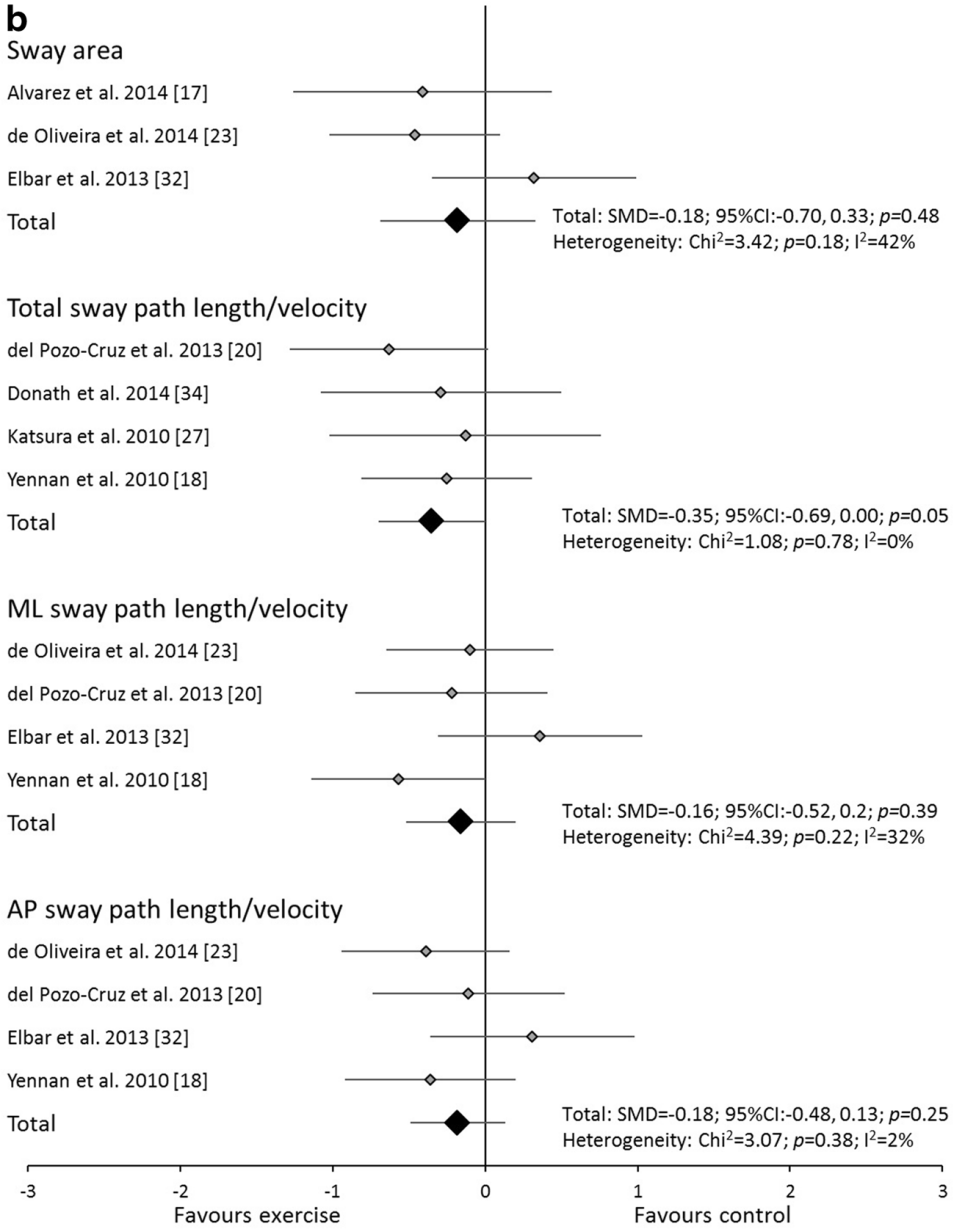
Forest plot of standardised mean differences (SMDs) and 95 % confidence intervals (CIs) for sway area and total, medio-lateral (ML) and anterior-posterior (AP) sway path length/velocity under **a** eyes open and **b** closed, double leg stance conditions for the resistance training interventions

**Fig. 4:**
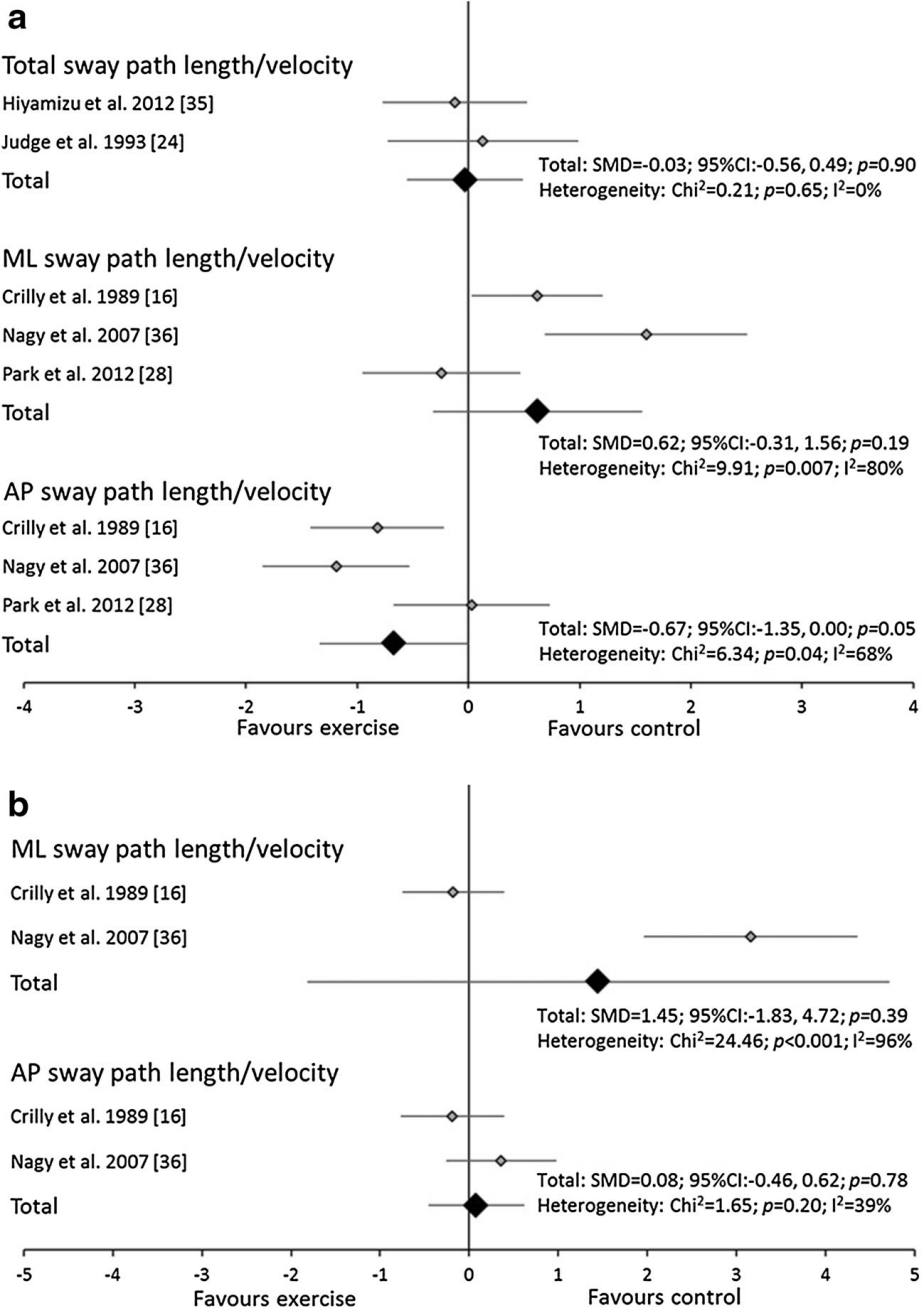
Forest plot of standardised mean differences (SMDs) and 95 % confidence intervals (CIs) for sway area and total, medio-lateral (ML) and anterior-posterior (AP) sway path length/velocity under **a** eyes open and **b** closed, double leg stance conditions for the multi-component training interventions

Resistance exercise interventions did not change any of the variables in either eyes open or closed conditions (Fig. 3a, b). Significant heterogeneity was observed only for AP sway path length/velocity in eyes open conditions for resistance exercise interventions. No study incorporating multi-component exercise reported sway area. Multi-component exercise interventions did not change any of the analysed variables in either eyes open or closed conditions (Fig. 4a, b). Significant heterogeneity was observed for AP and ML sway path length/velocity in eyes open conditions and ML sway path length/velocity in eyes closed conditions for multi-component exercise interventions.

## Discussion

The present systematic review identified a variety of static balance assessments and measurement protocols. Despite this variation in assessments and measurements, balance exercise interventions appear effective in improving the postural control of older adults as shown by the improvements of various postural control measures. In contrast, resistance or multi-component exercise interventions did not change any of the postural control measures.

Based on our meta-analyses, balance exercise interventions improve postural control, whereas other types of exercise intervention do not. However, this is in contrast with exercise interventions that are used to increase gait speed, where resistance, coordination and multi-component exercise were equally effective [38]. This suggests that postural control is a separate construct, compared to a more complex, dynamic task such as walking [38]. The findings of a recent systematic review confirm this view, and suggest that muscle strength and balance are individual neuromuscular components that need to be assessed and targeted separately [39]. The present meta-analyses also supports the view that strength training does not improve balance [40]. Nevertheless, the alterations in postural control are in agreement with a past systematic review showing improvements in balance performance of older adults following exercise interventions [5]. The present systematic review on COP measurements further extends this knowledge, and shows that a likely mechanism behind the improvements in balance performance is improved postural control.

A novelty of the present systematic review was the explicit focus on postural control rather than generic balance assessments (e.g. Berg Balance Scale), which enables a better understanding of the mechanisms for potentially improved balance performance. The effect of exercise interventions on sway area becomes more apparent when the balance task is performed with eyes closed compared with eyes open. A likely explanation is that postural control in eyes closed conditions relies solely on efferent neuromuscular and sensorimotor input, which can be improved with exercise [21, 41]. In contrast, postural control in eyes open conditions is influenced by visual input, which elicits a decreased sway area compared with eyes closed. Thus, sway area with eyes open conditions is unlikely to reflect the postural control mechanisms that are improved with exercise [42]. The improved efferent neuromuscular and sensorimotor function associated with balance exercise training allows the body to constrain postural sway to a smaller area and lower movement velocity, subsequently reducing the demand for large postural corrections. Overall, the meta-analyses provide a strong rationale for the use of balance assessments with eyes closed conditions, to evaluate the potential improvements in efferent neuromuscular and sensorimotor function.

The double leg stance was the most commonly used assessment, whereas other tasks (e.g. single leg stance, tandem stance) were less common. A likely explanation is the balance performance of the general older adult population, who are unlikely to be able to perform more complex tasks such as single leg stance for sufficient amount of time to record postural control [43]. The double leg stance was sufficient to show a decrease in total and AP sway path length following balance exercise interventions. In contrast, sway area with eyes open and ML sway path length with eyes closed were not altered following an exercise intervention. This could potentially be explained by the efficacy of balance exercise interventions and the nature of the exercises included. Potentially, balance exercises would favour improvements in muscles that act primarily to cause movement in the AP direction [24, 28] rather than those which act to control ML sway. This approach is understandable as the anatomy of the leg, in particular the ankle joint, means that there is a greater freedom of movement in the AP direction compared with the ML direction [44]. Alternatively, the majority of studies lacked a standardisation of stance width between trials and visits, which could have influenced the results [45]. Secondly, 11 [15, 17, 24–27, 29, 30, 33–35] of the 22 studies included in the meta-analyses did not report AP and ML sway characteristics separately, reporting only sway area or total sway path length. By overlooking this, it is possible that studies have missed important detail in the interpretation of postural control changes after exercise interventions. Thirdly, the effect size of 0.8 for ML sway path length was similar to the other measurements, except for sway area, where the effect sizes were considerably smaller. The ML sway path length also showed considerable heterogeneity (88 %). Thus it seems likely that the lack of a decrease in ML sway path length was due to the large heterogeneity, caused by inconsistencies in the data collection protocols.

There was substantial variability in the protocols of the studies included in the meta-analyses. A recent systematic review has suggested that averaging three to five trials of a minimum of 60 s and preferably 90 s in length is required to achieve good reliability for all traditional postural control measures [46]. Based on these recommendations none of the reviewed trials met the requirements for reliable assessment of postural control, which may have impacted upon the findings of all analysed variables. In addition, the sampling frequency used can also impact on the calculated variables, as can the signal processing applied. Measurements of sway velocity and area increase as sampling frequency increases [46]. It should therefore be ensured that sampling frequency meets the requirements of the Nyquist sampling theorem [11]. If a high sampling frequency is to be used, then a digital signal filter could be required to limit the inclusion of noise within the COP signal. Although signal filtering has the benefit of removing noise, it can result in the loss of the complex detail of the postural control system [47]; thus, it has been suggested that, depending on the study design and the outcome variable selected, a sampling frequency of 100 Hz be used in conjunction with a filter with a 10-Hz cut-off frequency [46].

There were several limitations in the present review. Firstly, the present meta-analyses revealed substantial heterogeneity between trials for the different postural control outcome variables. This may relate to the use of a cohort of older adults with varying health status and intervention characteristics (e.g. duration, frequency) [5], which may result in differing effect sizes for the same measurements between studies. Due to the low number of studies for each exercise type, it was impossible to determine whether the effectiveness of balance exercise to improve postural control depends on the participant’s characteristics (e.g. healthy or frail). In addition, a low number of included studies for each type of exercise intervention could have influenced the interpretation due to a lack of statistical power, in particular for resistance and multi-component exercise interventions, which had lower effect sizes and/or higher heterogeneity. Another limitation was the exclusion of non-force platform-based postural control outcome variables and outcome variables not related to sway area, sway path length and velocity. Future research could evaluate whether outcome variables such as the limits of stability or non-linear analysis are affected by exercise interventions. Finally, publication bias could have influenced the ability to retrieve randomised controlled trials reporting non-significant findings in relation to postural control.

## Conclusion

In conclusion, the systematic review demonstrated that only balance exercise can improve total and AP sway with eyes open and closed in community-dwelling older adults. Thus, postural control is altered by specific, targeted exercise interventions, but not by resistance or multi exercises. However, researchers should make various improvements to the design of future trials that include the assessment of postural control, as studies were often deficient in important considerations that influence study quality. A minimum duration of 60 s and at least three to five trials has been previously recommended. The absence of a change in ML sway could be due to lack of standardisation in measurement protocol. Based on the present meta-analyses, it is recommended that future studies measure changes in postural control via the reporting of movement in the AP direction as a minimum, and that double leg stance with eyes closed appears to be the preferred static balance assessment.

## Electronic supplementary material

Below is the link to the electronic supplementary material.

Supplementary material 1 (DOCX 36 kb)
