# Supplementary material for: Effectiveness of Exercise Interventions to Improve Postural Control in Older Adults: A Systematic Review and Meta-Analyses of Centre of Pressure Measurements
Source: Sports Med. 2016 May 31;47(1):101–12. doi: 10.1007/s40279-016-0559-0 (PMC5215248; doi:10.1007/s40279-016-0559-0)
Supplement: Supplementary file 1 — Supplementary material 1 (DOCX 36 kb) [file 40279_2016_559_MOESM1_ESM.docx]

**Electronic Supplementary Material**

Electronic Supplementary Material Table S1. Population demographics and intervention details of the included trials.

| Reference | Population Health Status | Control Demographics | | Experimental Demographics | | Intervention Details | | | | |
| --- | --- | --- | --- | --- | --- | --- | --- | --- | --- | --- |
|  |  | N(female) | Age (yrs) | N(female) | Age (yrs) | Control Exercise | Experimental Exercise | Duration (wks) | Frequency (session/wk) | Session Length (mins) |
| Alvarez et al. 2014 [17] | Institutionalised | 11(9) | 86±7 | 11(8) | 84±3 | ADL | Vibration | 8 | 3 | 12-17 |
| Crilly et al. 1989 [16] | Institutionalised | 24(24) | - | 23(23) | - | ADL | Strength + balance | 12 | 3 | 15 -35 |
| Del Pozo-Cruz et al. 2013 [20] | Diabetes | 20(10) | 66±10 | 19(10) | 71±8 | ADL | Vibration | 12 | 3 | 8-12 |
| de Oliveria et al. 2014 [23] | Healthy | 20(20) | 69±4 | 20(20) | 69±3 | Gymnastics | Water gymnastic | 24 | 2 | 60 |
| Donath et al. 2014 [34] | Healthy | 12(6) | 70±6 | 13(6) | 69±3 | ADL | Stair climbing | 8 | 3 | Varied |
| Elbar et al. 2013 [32] | Healthy | 17(-) | 69±4 | 18(-) | 69±5 | ADL | Water based | 12 | 2 | 40 |
| Hiyamizu et al. 2012 [35] | Healthy | 19(16) | 71±4 | 17(10) | 72±5 | ADL | Strength +  balance | 12 | 2 | 60 |
| Judge et al. 1993 [24] | Healthy | 9(9) | 68±4 | 12(12) | 67±2 | Flexibility | Strength +  Tai chi | 26 | 3 | - |
| Kaneda et al. 2008 [33] | Healthy | 15(13) | 60±4 | 15(13) | 60±4 | ADL | Water based | 12 | 2 | 80 |
| Katsura et al. 2010 [27] | Healthy | 8(5) | 70±5 | 12(11) | 68±4 | Water based | Water with resistance | 8 | 3 | 90 |
| Lai et al. 2013 [29] | Healthy | 15(8) | 74±4 | 15(9) | 70±3 | ADL | Balance - video game | 6 | 3 | 30 |
| Lee and Song. 2012 [19] | Diabetes | 27(19) | 73±4 | 28(20) | 74±5 | ADL | Balance - Virtual reality game | 10 | 2 | 50 |
| Lee et al. 2013 [22] | Diabetic peripheral neuropathy | 18(10) | 75±5 | 18(11) | 74±5 | ADL | Balance | 6 | 2 | 60 |
| Lelard et al. 2010 [25] | Healthy | 14(10) | 77±4 | 14(11) | 76±5 | Balance | Tai chi | 12 | 2 | 30 |
| Nagai et al. 2012 [30] | Healthy | 24(22) | 81±6 | 24(20) | 81±6 | ADL | Balance | 8 | 2 | 40 |
| Nagy et al. 2007 [36] | Healthy | 9(6) | 79±1 | 10(7) | 73±4 | ADL | Strength +  balance | 8 | 2 | 45 |
| Nicholson et al. 2014 [31] | Healthy | 13(12) | 65±5 | 15(11) | 66±4 | ADL | BodyBalance | 12 | 2 | - |
| Ni et al. 2014 [15] | Fallers | 15(9) | 77±7 | 13(13) | 73±5 | Balance | Tai Chi | 12 | 2 | 60 |
| Park et al. 2012 [28] | Healthy | 15(8) | 77±7 | 16(9) | 76±8 | Therapeutic exercise | Therapeutic + jumping | 4 | 5 | 40 |
| Pirouzi et al. 2014 [37] | Healthy | 15(2) | 71±6 | 14(10) | 70±5 | ADL | Treadmill | 4 | 3 | 30 |
| Pluchino et al. 2012 [26] | Healthy | 14(9) | 72±7 | 14(8) | 69±6 | Balance | Tai chi | 8 | 2 | 60 |
| Song et al. 2011 [21] | Diabetic peripheral neuropathy | 19(11) | 73±5 | 19(7) | 72±5 | ADL | Balance + health advice | 8 | 2 | 60 |
| Yennan et al. 2010 [18] | Osteoarthritis | 25(25) | 66±4 | 25(25) | 65±4 | Land - strength | Water based | 6 | 6 | 65 |

Abbreviations: ADL: normal activities of daily living; - indicates that no relevant information was reported

Electronic Supplementary Material Table S2. Details of conditions, tasks, and data collection and processing details used to assess postural control in the included trials.

| Reference | Conditions | Task | Number x duration of trials per condition | Equipment | Sampling Freq (Hz) | COP signal Processing | Outcome Variables (units) |
| --- | --- | --- | --- | --- | --- | --- | --- |
| Alvarez et al. 2014 [17] | EO, EC, cognitive dual task | DL: quiet stance | 3 x 30 s | Force platform | 1000 | - | Sway area (cm^2^) |
| Crilly et al. 1989 [16] | EO, EC | DL: quiet stance | 2 x 20 s | Force platform | - | - | Total, AP and ML sway path lengths (mm) |
| Del Pozo-Cruz et al. 2013 [20] | EO, EC | DL: quiet stance, narrow stance | 3 x 30 s | Nintendo Wii balance board | 100 | Filtered – 8 Hz | Total, AP and ML sway path lengths (cm) |
| de Oliveria et al. 2014 [23] | EO, EC | DL: quiet stance, semi-tandem,  SL: quiet stance | 3 x 30 s | Force platform | 100 | Filtered – 35 Hz | Sway area (mm^2^), AP and ML sway path velocity (mm/s) |
| Donath et al. 2014 [34] | EO, EC | DL: quiet stance  SL: quiet stance | 3 x 30 s | Force platform | 40 | Filtered – 10 Hz | Total sway path length (mm) |
| Elbar et al. 2013 [32] | EO, EC | DL: quiet stance | 10x30s | Force platform | 100 | - | Sway area (mm^2^), AP and ML sway path lengths (mm) |
| Hiyamizu et al. 2012 [35] | EO, EC, cognitive dual task | DL: quiet stance | 1 x 30 s | Force platform | 50 | - | Total sway path length (cm) |
| Judge et al. 1993 [24] | EO, EC | DL: quiet stance  SL: quiet stance | 3 x 8 s | Force platform | 100 | - | Total sway path length (mm) |
| Kaneda et al. 2008 [33] | EO | DL: quiet stance | 1 x 30 s | Force platform | - | - | Sway area (cm^2^), total sway path length (cm) |
| Katsura et al. 2010 [27] | EO, EC | DL: quiet stance | - | Force platform | - | - | Total sway path length (cm) |
| Lai et al. 2013 [29] | EO, EC | DL: quiet stance | 1 x 75 s | Force platform | 15 | - | Sway area (mm^2^), total sway path length velocity (mm/s) |
| Lee and Song 2012 [19] | EO, EC | DL: quiet stance | 3 x 30 s | Force platform | - | - | Total, AP and ML sway path lengths (cm) |
| Lee et al. 2013 [22] | EO, EC | DL: quiet stance | 3 x 30 s | Force platform | - | - | AP and ML sway path velocity (mm/s) |
| Lelard et al. 2010 [25] | EO, EC | DL: quiet stance | 3 x 25.6 s | Force platform | 40 | - | Sway area (mm^2^), total sway path length (mm), total sway path velocity (mm/s), velocity variance (%) |
| Nagai et al. 2012 [30] | EO | DL: quiet stance | 1 x 10 s | Force platform | 20 | Filtered – 6 Hz | Sway area (cm^2^) |
| Nagy et al. 2007 [36] | EO, EC | DL: quiet stance | 1 x 20 s | Force platform | 16 | Filtered – 10 Hz | AP and ML sway path lengths (mm), high, medium and low frequency power |
| Nicholson et al. 2014 [31] | EO, EC | DL: quiet stance, narrow stance | 2 x 30 s | Force platform | 50 | Filtered – 10 Hz | ML sway path length (mm) |
| Ni et al. 2014 [15] | EO, EC | DL: quiet stance | 3 x 10 s | Force platform | - | - | Sway area (cm^2^), average and max displacements (cm), max and min velocity (cm/s) |
| Park et al. 2012 [28] | EO, EC | DL: quiet stance | 3 x 30 s | Force platform | 5 | - | AP and ML sway path length (mm) |
| Pirouzi et al. 2014 [37] | EO | DL: quiet standing, tandem, quiet on foam | 3 x 30 s | Force platform | 120 | Filtered – 6 Hz | AP and ML sway path length (mm), Total, AP and ML sway velocity (mm/s) |
| Pluchino et al. 2012 [26] | EO | DL: quiet stance | 3 x 10 s | Force platform | - | - | Sway area (m^2^), average and max displacements (m), max and min velocity (m/s) |
| Song et al. 2011 [21] | EO, EC | DL: quiet stance | 3 x 30 s | Force platform | - | - | Total, AP and ML sway path lengths (cm) |
| Yennan et al. 2010 [18] | EO | DL: quiet stance  SL: quiet stance | 2 x 30 s | Force platform | - | - | Sway area (mm^2^), AP and ML sway path length (mm) |

COP: Centre of pressure DL: double leg stance, SL: single leg stance, EO: eyes open, EC: eyes closed; - indicates that no relevant information was reported
